# Surveillance in easy to access population subgroups as a tool for evaluating malaria control progress: A systematic review

**DOI:** 10.1371/journal.pone.0183330

**Published:** 2017-08-16

**Authors:** Sanie S. S. Sesay, Emanuele Giorgi, Peter J. Diggle, David Schellenberg, David G. Lalloo, Dianne J. Terlouw

**Affiliations:** 1 Malawi-Liverpool-Wellcome Trust Clinical Research Programme, Blantyre, Malawi; 2 Liverpool School of Tropical Medicine, Liverpool, United Kingdom; 3 Medical School, Lancaster University, Lancaster, United Kingdom; 4 Institute of Infection and Global Health, University of Liverpool, Liverpool, United Kingdom; 5 London School of Hygiene and Tropical Medicine, London, United Kingdom; Tulane University School of Public Health and Tropical Medicine, UNITED STATES

## Abstract

**Background:**

The need for surveillance systems generating targeted, data-driven, responsive control efforts to accelerate and sustain malaria transmission reduction has been emphasized by programme managers, policy makers and scientists. Surveillance using easy-to-access population subgroups (EAGs) may result in considerable cost saving compared to household surveys as the identification and selection of individuals to be surveyed is simplified, fewer personnel are needed, and logistics are simpler. We reviewed available literature on the validation of estimates of key indicators of malaria control progress derived from EAGs, and describe the options to deal with the context specific bias that may occur.

**Methods:**

A literature search was conducted of all documents reporting validation of estimates of malaria control indicators from EAG surveys before the 31^st^ of December 2016. Additional records were identified through cross-reference from selected records, other applicable policy documents and grey literature. After removal of duplicates, 13, 180 abstracts were evaluated and 2,653 eligible abstracts were identified mentioning surveillance in EAGs, of which 29 full text articles were selected for detailed review. The nine articles selected for systematic review compared estimates from health facility and school surveys with those of a contemporaneous sample of the same population in the same geographic area.

**Results:**

Review of the available literature on EAGs suitable for surveillance of malaria control progress revealed that little effort has been made to explore the potential approach and settings for use of EAGs; and that there was wide variation in the precision of estimates of control progress between and within studies, particularly for estimates of control intervention coverage. Only one of the studies evaluated the geospatial representativeness of EAG samples, or carried out geospatial analyses to assess or control for lack of geospatial representativeness. Two studies attempted to measure the degree of bias or improve the precision of estimates by controlling for bias in a multivariate analysis; and this was only successful in one study. The observed variability in accuracy of estimates is likely to be caused by selection and/or information bias due to the inherent nature of EAGs. The reviewed studies provided insight into the design and analytical approaches that could be used to limit bias.

**Conclusion:**

The utility EAGs for routine surveillance of progress in malaria control at the district or sub-district programmatic level will be driven by several factors including whether serial point estimates to measure transmission reduction or more precise geospatial distribution to track ‘hot-spots’ is required, the acceptable degree of precision, the target population, and the resources available for surveillance. The opportunities offered by novel geostatistical analyses and hybrid sampling frames to overcome bias justify a renewed exploration of use of EAGs for malaria monitoring and evaluation.

## Background

The need for surveillance systems that inform accelerated and sustained control efforts to accelerate and sustain malaria transmission reduction has been emphasized by programme managers, policy makers and scientists. A key element of these surveillance systems will be their cost and whether they can easily be integrated with current malaria control activities. Routine health facility-based passive case reporting, for example through Health Management Information Systems (HMISs), has been and continues to be at the forefront of malaria surveillance [1, 2]. A well-functioning HMIS will provide regular data from all health facilities nationally allowing accurate measurement of malaria control progress across the healthcare system. This has largely not been the case for most HMISs in malaria endemic countries, with problems like incomplete reporting and lack of diagnostic confirmation being comparatively common [3, 4]. Malaria indicator surveys (MISs) provide single cross-sectional national assessments of disease burden [5], but are usually expensive and logistically demanding to undertake. The goal of MISs is to generate nationally representative estimates and are thus not powered to detect local-level variability[6, 7]. The interval between serial MISs also affect their direct relevance for monitoring short- and medium-term trends in malaria control progress. Supplementary approaches are thus needed to provide timely estimates of malaria control progress at the district and sub-district level, complementary to current malaria surveillance systems, particularly as malaria transmission intensity falls and its distribution becomes more localized [8].

Representative subsets of the population or disease at-risk groups routinely assemble at easily accessible locations (e.g. schools or health facilities) making them logistically attractive to sample [9]. Alternatively, representative subgroups or the whole population of interest may be easily accessible during public health intervention activities such as mass drug administration and catch-up vaccination campaigns [10]. The opportunistic nature of surveillance in the so called Easy Access Groups (EAGs) could thus save costs by simplifying the identification and selection of individuals to be surveyed, requiring simplified logistics and fewer study personnel compared to household surveys [9, 11]. Evidence from school surveys indicate that EAGs are suitable for surveillance when they are potentially representative of an at-risk stratum of the population [9]. However, there are concerns about the inherent biased nature of such a sample, as such non-probability samples depend on natural systems of selection which are likely to result in the selection of a non-representative sample of the population of interest [12]. In this systematic review, we studied the available literature on the validation of estimates of key indicators of malaria control progress [13] derived from EAGs, focusing on EAGs that may be suitable for surveillance at the district or (sub)district level.

## Methods

### Search strategy

We searched EMBASE® (EMBASE, Medline, EMBASE Classic), PubMed® and ScienceDirect® bibliographic databases without language restrictions from inception to 31^st^ December 2016 for articles with the following search terms in their keywords, title or abstract: "malaria" AND "survey”; or “malaria” AND “surveillance”, or "malaria" AND “monitoring” AND “evaluation”; or "malaria" AND "transmission" AND "measurement. We also searched the online WHO document centre [14] for relevant policy documents and for grey literature from the WHO historical documents database on malaria (1947–2000) [15]. We also included pertinent articles that were not picked up by our search from other sources including recommendation from key experts in the field of malaria surveillance.

We compiled the results into a searchable database in EndNote X8.0.1 (Thomson Reuters). We searched this database for abstracts detailing validation of estimates from EAGs predetermined to be most suitable for routine malaria surveillance at the (sub)district-level by a review of historical evidence of previous use for malaria surveillance. We also added EAGs that had not been previously used for malaria surveillance but demonstrated this potential through surveillance of other diseases. Selected EAGs were further validated by examination against general criteria used to evaluate the suitability of a surveillance system [16], adapted to malaria surveillance (Table 1). Based on our review we postulated that the following EAGs were suitable for the routine surveillance of malaria control progress (Table 2):

1. School children
2. Health facility attendees, including:
  - All health facility attendees including accompanying persons
  - Children coming for sick or routine “well” child visits
  - Women attending ANC or coming for delivery
3. Population targeted by public health intervention campaign such as mass drug administration
4. Population attending rural community markets

**Table 1:** Criteria evaluating the suitability of EAGs for malaria surveillance.

Attribute	Definition
Suitability	
Usefulness	Contributes to understanding the epidemiology of malaria in the study area. Generates a suitable prompt public health response by impacting policies and/or control response.
Cost-effective	The direct and indirect costs should be justifiable in relation to the benefits attained.
Quality	
Sensitivity	The ability of the surveillance system to measure presence of relevant impact indicators.
Specificity	The ability of the surveillance system to identify the absence of relevant impact indicators.
Representativeness	Accurately reflects the spatio-temporal distribution of key health events and uptake of public health control measures in the population or key at-risk groups.
Timeliness	Ability to provide timely estimates of key health events to guide control efforts.
Simplicity	Easy to understand and implement.
Flexibility	Ability to be easily adapted to include new or emerging problems, other health events, population sub-groups or key disease at-risk groups.
Acceptability	Willingness of persons conducting surveillance and those providing data to generate accurate, consistent and timely data. Acceptability to other key stakeholders, the community, health planners, donors, etc.

**Table 2:** Advantages and disadvantages of EAGs suitable for malaria surveillance.

EAG	Advantages	Disadvantages
• School children	• Age range of primary school children in Africa of 5 to 14 years captures the *Pf*PR peak [84, 85] • Allows direct measurement of impact of malaria control interventions targeted at school children [86] • Extensively assessed historically [17, 87] and at the district and sub-district level [11, 88]	Substantial variations in primary school enrolment rates between different regions in sub-Saharan Africa [86]
• Health facility attendees		
○ All health facility attendees	• Less susceptible to problems of HMISs such as incomplete reporting and lack of diagnostic confirmation [3, 89]	• Representativeness of data on control progress from health facilities surveys will depend largely on health facility utilization rates [52, 90, 91]
○ Health facility attendee sub-groups		
■ Children coming for sick or “well” child visits	• Mostly infants which are a sensitive group to measure malaria transmission [92] • Can be used to directly assess coverage where immunization clinics have been used to distribute malaria control interventions [93]	• Blood sampling is required may have ethical considerations and may cause poor acceptance especially in children coming for well child visits • Same considerations for representativeness as above
■ Women attending ANC or coming for delivery	• Pregnant women are more susceptible to malaria regardless of endemicity making them a sensitive group to measure malaria transmission [19, 94] • Parity specific susceptibility suggest primigravidae are an even more sensitive at-risk sub-group [95–97] • ANC attendance is high and most women attend ANC at least once during their pregnancy [57] • *Pf*PR at the first antenatal booking is likely to reflect population transmission pressure as these women are yet to receive control interventions targeted at malaria in pregnancy [98] • Blood sampling requirement at first ANC visit and at delivery can be used to assess *Pf*PR and APR	• No integrated strategic approach to surveillance of malaria control in pregnancy currently so indicators need to be validated [99] • Relationship between the prevalence of peripheral and placental parasitaemia in pregnant women and that of the population is poorly understood [100] • Women with lower SES in developing countries are less likely to deliver in health facilities and this affects representativeness [101]
• Population targeted by public health intervention/campaign	• Most of the population or at-risk group is available for sampling • Mass ITN distribution, national immunization days (NIDs), mass drug administration (MDA) and surveys for NTDs offer excellent opportunities to integrate malaria surveillance, and has been assessed with MDA for filariasis [20] and surveys for trachoma [102]	• Unlikely to be a source of continuous data
• Population attending rural community markets	• Rural markets in large, centrally place towns offer an opportunity to survey a large potentially representative sample of the adult community of the surrounding area involving all social strata, and has not been assessed for malaria surveillance but in other diseases [103][42][104]	• Needs to be validated for malaria surveillance, and in urban settings

We then searched the EndNote database for articles with the following keywords in their abstract:

1. “school” AND “survey”, “school AND “surveillance”, “school” AND “monitoring” AND “evaluation”, and “school” AND “transmission” AND “measurement”
2. “health” AND “facility” OR “centre” AND “survey”, health” AND “facility” OR “centre” AND “surveillance”, “health” AND “facility” OR “centre” AND “monitoring” AND “evaluation”, and “health” AND “facility” OR “centre” AND “transmission” AND measurement
3. “antenatal clinic” AND “survey”, “antenatal clinic” AND “surveillance”, “antenatal clinic” AND “monitoring” AND “evaluation”, “antenatal clinic” AND “transmission” AND “measurement”, “pregnancy” OR “delivery” AND “survey”, “pregnancy” OR “delivery” AND “surveillance”, “pregnancy” OR “delivery” AND “monitoring” AND “evaluation”, and “pregnancy” OR “delivery” AND “transmission” AND “measurement”
4. “market” AND “survey”, “market” AND “surveillance”, “market” AND “monitoring” AND “evaluation”, and “market” AND “transmission” AND “measurement”
5. “public health” AND “intervention” OR “campaign” AND “survey”, “public health” AND “intervention” OR “campaign” AND “surveillance”, “public health” AND “intervention” OR “campaign” AND “monitoring” AND “evaluation”, and “public health” AND “intervention” OR “campaign” AND “transmission” AND “measurement”

### Inclusion criteria

**Fig 1:**
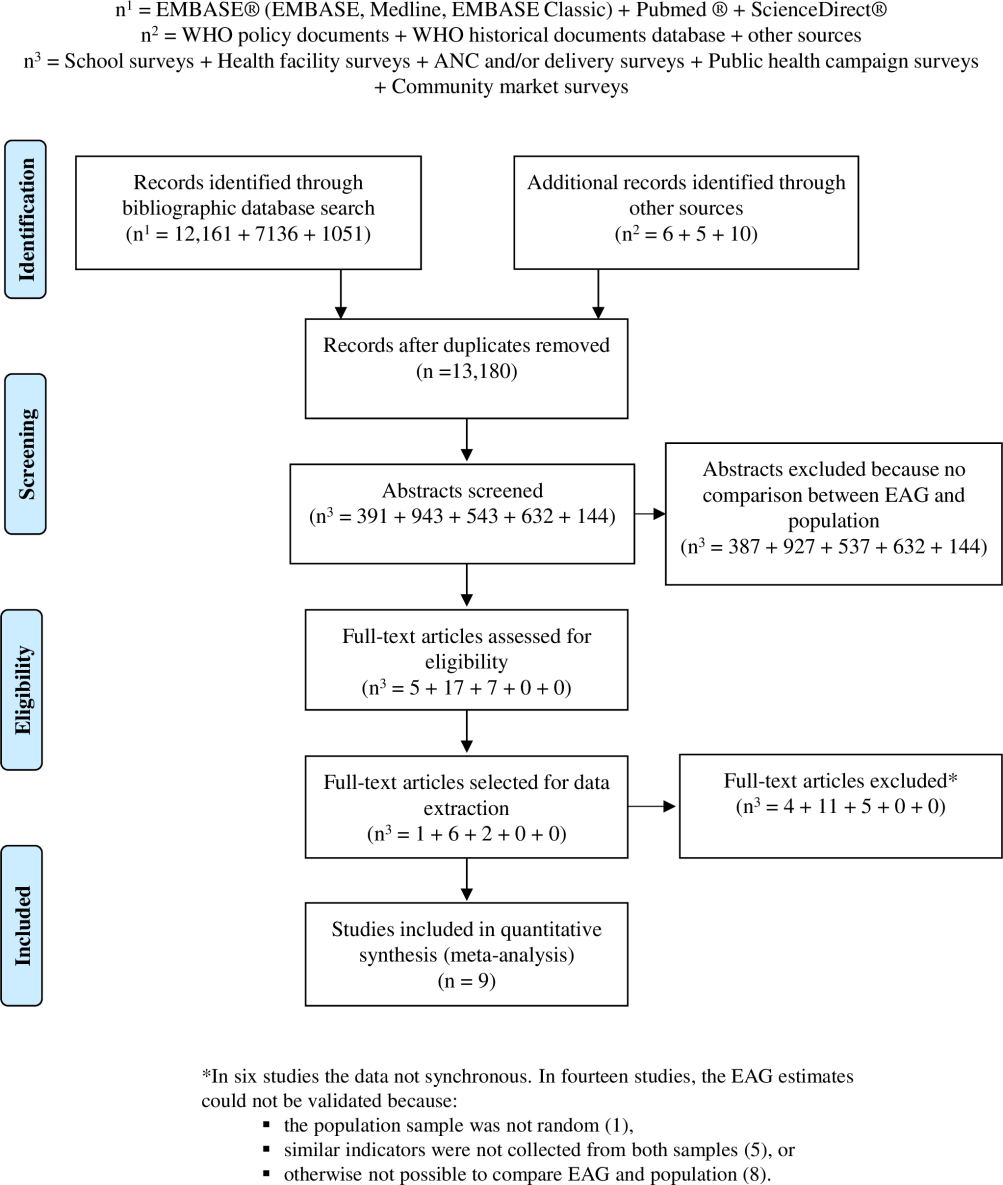
PRISMA flow diagram for studies comparing estimates between EAG and population surveys.

A total of 13, 180 records were compiled into a searchable database, at which the key word search resulted in the selection of 2,653 eligible abstracts for further review. These abstracts were reviewed for specific mention of the comparison of malaria indicator estimates from an EAG sample with population sample (Fig 1) and 29 articles were selected for full text review.

### Exclusion criteria

We searched for the full text of the selected 29 publications, and excluded studies in which estimates of malaria control indicators from EAGs were not compared to a contemporaneous random population sample from the same geographic area. Since the distribution of *Plasmodium falciparum* infection in the population is determined by environmental factors that influence the density of competent anopheline mosquitos, location-specific vector behaviour, and human factors like at-risk status (e.g. age and pregnancy) and behaviour (e.g. ITN use) that increase exposure to infectious mosquito bites [17–19]; to increase the accuracy of EAG *Pf*PR estimates, we excluded all studies that did not compare EAG samples to population samples from the same age or other at-risk stratum.

### Selection of studies

Twenty of the twenty-nine studies selected for full review satisfied one or more exclusion criteria and were not included in the systematic review (Fig 1). Six of the studies were excluded because the data collected was not sufficiently synchronous between the EAG and population sample [20–25]. In fourteen studies the validity of EAG estimates could not be determined either because the population sample was not random [26], the same indicators were not collected from both samples [27–31], or both samples were otherwise not comparable [32–39]. In the nine selected studies, information was recorded on the type of EAG, comparator population, sampling frame, sampling methodology, sample size and sampling units. Data on the first author, year of survey, survey site, year of publication, malaria transmission intensity (e.g. *Pf*PR), and estimates of control progress were extracted for the systematic review.

### Definitions

*Anaemia prevalence rate (APR)*–Proportion of the population with a haemoglobin measurement of <8 g/dL.

*Antibody prevalence rate (AbPR)*–Proportion of the population seropositive to defined malaria antigens.

*Household bed net ownership*–Proportion of households with at least one bed net.

*Household ITN ownership–*Proportion of households with at least one ITN.

*Individual bed net use*–Proportion of population that slept under a bed net the previous night.

*Individual insecticide treated bed net (ITN) use*–Proportion of population that slept under an ITN the previous night.

*Indoor residual spraying (IRS) coverage*–Proportion of households sprayed with IRS in the past 12 months.

*Plasmodium falciparum prevalence rate (PfPR)*–Proportion of the population with malaria infection detected by rapid diagnostic test (RDT), microscopy or polymerase chain reaction (PCR).

*Sick child visit–*Health facility visits during childhood for an illness episode.

*Well child visit–*Routine health facility visits that occur during childhood that may include immunizations, growth and development assessments, physical examination and other health risk assessments.

### Statistical analysis

Data analysis was done using Stata version 13.1® (StataCorp, Texas, USA). Using the presented data from tables in the selected publications, we calculated point estimates of control progress indicators derived from EAGs and compared that to estimates from contemporaneous population samples. In one publication [40], due to absence of the numerator, we derived the numerator from the reported rates and the denominator, and then calculated point estimates and corresponding 95% confidence intervals. Where surveys were repeated either seasonally or after a specific period [41, 42], we presented these estimates separately to account for seasonal or temporal effect respectively. We assessed the degree of accuracy in estimates derived from EAG samples by examining the absolute difference in prevalence difference and corresponding 95% confidence intervals and Pearson’s χ2 p values. Mean prevalence was derived for the overall individual level estimates from the EAG and population samples. The estimates for *Pf*PR were derived individually for each method of detection of parasitaemia e.g. blood film, rapid diagnostic test. Malaria endemicity was classified according to the revised Global Malaria Eradication Program classification [43]. Due to the inherent differences in EAGs and the paucity of studies, we did not derive pooled estimate effects for each malaria control indicator. To evaluate the effect of population coverage of control interventions and transmission intensity on the validity of EAG estimates of control interventions and *Pf*PR respectively, where possible, we correlated the prevalence difference with the population prevalence. We also evaluated the potential for any of the EAG samples to misclassify an area into the wrong malaria endemicity category by comparing the classification of each area by population *Pf*PR to that from EAG estimates.

## Results

### Description of studies

Nine studies were included in the systematic review (Table 3), all of which were from sites with intense stable or moderate stable malaria transmission. Six studies assessed the accuracy of estimates from health facilities [40–42, 44–46], two studies assessed the accuracy of estimates from school surveys [47, 48], and one study assessed the accuracy of estimates from antenatal clinics [49]. Three studies compared estimates from children less than 5 years old [40, 42, 44], two studies compared estimates from older children [47, 48], and four studies compared estimates from all presenting individuals at health facilities (including ANC) regardless of age [41, 45, 46, 49].

**Table 3:** Description of studies comparing estimates between EAG and population surveys.

Study	Study year(s)	Country	Geographic unit of comparison	Site(s)	Malaria endemicity*	EAG			Population		
						Participants	Sampling methods	Sampling Units; No. sampled	Participants	Sampling methods	Sampling Units; No. sampled
Briand et al [49]	2014	Laos	Region	Salavan	Moderate stable	Women delivering in health facilities	Successive	2 hospitals n = 331	Pregnant women living in households	Random selection of villages; pregnant women invited to participate	30 villages: n = 205 pregnant women
Gahutu et al. 2011 [44]	2012	Rwanda	Sub-district or local	Butare	Moderate stable	Children < 5 years coming for sick child visits	Successive	1 hospital; n = 101 1 health centre; n = 103	Children < 5 years living in households	Stratified random	24 villages; n = 545 households
Hetzel et al [45]	2008/9	Papua New Guinea	Region	Momase and Highlands	Moderate stable	All patients attending health facility past 3 days	Successive	3 health centres n = 1304	All individuals living in households older than 5 months	Random selection of villages from health facility catchment area; random selection of households	3–4 villages from each health facility catchment area; n = 1967
	2009/10					All patients attending health facility with a history of fever in the past 3 days		3 health centres n = 677	All individuals living in households older than 5 months	Random selection of villages from health facility catchment area; random selection of households	3–4 villages from each health facility catchment area; n = 1986
Karyana et al [46]	2004/5	Papua New Guinea	District	Mimika	Moderate stable	All patients attending health facilities	Routine HMIS surveillance	1 hospital; n = 186040 14 primary health clinics; n = 253987	All individuals living in households	Random selection of households by three-stage cluster sampling	800 households
Mathanga et al. 2010 [42]	2005	Malawi	District	Phalombe Blantyre Chiradzulu Mwanza Lilongwe Rumphi	Moderate stable	Well children 6–30 months attending EPI clinics	Systematic	12 EPI clinics; n = 1637	Children aged 6–30 months living in households	Stratified random, probability proportional to enumeration area	30 enumeration areas; n = 926 households
	2008	Malawi	District	Phalombe Blantyre Chiradzulu Mwanza Lilongwe Rumphi	Moderate stable	Well children 6–30 months attending EPI clinics	Systematic	12 EPI clinics; n = 1909	Children aged 6–30 months living in households	Modified EPI cluster survey	30 enumeration areas; n = 4565 households
Ndyomugyenyi et al. 2007 [47]	2005	Uganda	District	Hoima	Intense stable	Primary school children ≥ 10 years	Purposeful	39 primary schools; n = 3602	Household heads or spouses	Stratified random	39 villages; n = 2798 households
Oduro et al. 2011 [41]	2008	Gambia	Country	Albreda Kaur Yorobawol Gambisara Bureng Gunjur	Moderate stable-	All patients attending health facilities	Successive	6 health centres; n_1_ = 4543 (rainy/post rainy season) n_2_ = 4101 (dry season)	All villagers	Age-stratified random	18 villages (3 from each catchment area); n_1_ = 3870 households (rainy/post rainy season) n_2_ = 3716 households (dry season)
Skarbinski et al. 2008 [40]	2005	Tanzania	Region	Lindi	Intense stable-	Children < 5 years coming for sick and well child visits	Stratified cluster sampling (Lindi)	5 randomly chosen health facilities; n_1_ = 444 (well child visits) n_2_ = 193 (sick child visits)	Household members	Stratified random, probability proportional to enumeration area (Lindi)	22 enumeration areas; n = 574 households
			District	Rufiji			All (Rufiji) on day of survey	4 health centre; n_1_ = 911 (well child visits) n_2_ = 522 (sick child visits)		Simple random (Rufiji)	N/A; n = 673 households
Stevenson et al. 2013 [48]	2010	Kenya	District	Rachuonyo Kisii	Moderate stable-	Primary school children in classes 2–6	46 government primary schools; n = 4888	Gender-stratified random sampling	All children > 6 months living in compounds	Simple random sampling, within 600m of each school	N/A; n = 3472 households

*Malaria endemicity:

Moderately stable endemicity: PfPR = 5.1–39.99% i.e. hypo-mesoendemic

Intensely stable endemicity: PfPR>40% i.e. hyper-holoendemic

Unstable endemic: PfPR <5%.

### Comparison of estimates

#### Estimates of coverage of control interventions

Seven studies assessed the accuracy of estimates of coverage of control interventions. (Table 4) [40–42, 44, 47–49]. The estimates of coverage of different control interventions derived from EAGs were significantly higher than those of the population in three studies [40, 44, 47], except for the estimates of household ITN ownership which was concordant with the population in one of these studies [47]. In three studies, estimates of control intervention coverage were significantly lower in EAGs [41, 48, 49]. In one study, estimates derived from parents/guardians of children aged 6–30 months coming for well child visits in Malawi were concordant in the first year of survey (2005) but significantly higher in the second survey (2008) [42]. In 2005, the estimates of individual bed net use derived from this EAG (PR = 41.0%, 95% CI 38.9%, 47.4%) were slightly lower than that in the same age stratum in the population (PR = 45.4%, 95% CI 39.0%, 51.7%, p = 0.0339), though this difference is not significant due to overlapping confidence intervals. Similarly, the estimate of individual ITN use derived from the EAG in the same survey (PR = 36.7%, 95% CI 31.1%, 42.4%) was not significantly different from that of the population (PR = 41.0%, 95% CI 34.1%, 40.5%, p = 0.0311). The study by Stevenson et al [48] investigated the concordance in school and catchment area-based estimates of control intervention coverage across a range of circumferential distances around each school. Estimates of individual bed net use derived from school children living 601-1000m (PR = 31.3%, 95%CI 29.1%, 33.5%) and >1000m (PR = 32.9%, 95%CI 29.1%, 33.5%) from the school were not significantly different from those from school children within 600m of the school (PR = 33.4%, 95% CI 31.2%, 35.6%), indicating that inaccuracy remained relatively constant with changes in circumferential area within the school’s catchment area. In the same study, estimates of IRS coverage from school children living 601-1000m (PR = 70.7%, 95%CI 68.5%, 72.8%) and >1000m (PR = 72.9%, 95%CI 68.5%, 72.8%) from the school were not significantly different from those from school children within 600m (PR = 68.3%, 95%CI 66.1%, 70.4%) of the school again indicating the inaccuracy was not affected by circumferential area within the school’s catchment area.

**Table 4:** Comparison of estimates of coverage of control interventions between EAGs and the population.

Control intervention coverage	Type of EAG survey	EAG survey		Population survey		Fisher’s exact p-value
		Events (n/N)	Percentage prevalence (95% CI)	Events (n/N)	Percentage prevalence (95% CI)	
**Household bed net ownership**						
Briand et al						
• Salavan, Laos	ANC	307/331	92.8 (90.0; 95.5)	204/205	99.5 (98.5; 100.0)	<0.001
Ndyomugyeni et al						
• Hoima, Uganda	School	1261/3602	35.0 (33.5; 36.6)	867/2798	30.9 (29.3; 32.7)	<0.001
Skarbinkski et al						
• Lindi, Tanzania	Health Facilities	506/637	79.4 (76.3; 82.6)	163/354	46.1 (40.9; 51.2)	<0.001
• Rufiji, Tanzania	Health Centre	1195/1433	83.4 (81.5; 85.3)	337/455	74.1 (70.0; 78.1)	<0.001
**Household ITN ownership**						
Ndyomugyeni et al						
• Hoima, Uganda	School	814/3602	22.5 (21.2; 24.0)	629/2798	22.5 (20.9; 24.0)	0.9759
**Individual bed net use**						
Briand et al						
• Salavan, Laos	ANC	305/331	92.2 (89.3; 95.0)	204/205	99.5 *98.5; 100.0)	<0.001
Gahutu et al						
• Butare, Rwanda	Health Centre	71/102	69.6 (60.7; 78.5)	286/543	52.7 (48.5; 56.9)	0.0016
• Butare, Rwanda	Hospital	74/102	72.6 (63.9; 81.2)	286/543	52.7 (48.5; 56.9)	<0.001
Mathanga et al						
• Malawi^d^	Health Centre	671/1637	41.0 (38.6; 43.4)	420/926	45.4 (42.2; 48.6)	0.0339
• Malawi^e^	Health Centre	1067/1909	55.9 (53.7; 58.1)	1899/4565	41.6 (40.2; 43.0)	<0.001
Oduro et al						
• Gambia (2005)	Health Centre	3568/4543	78.5 (77.3; 79.7)	3348/3870	86.5 (85.4; 87.6)	<0.001
• Gambia (2008)	Health Centre	2848/4101	69.5 (68.0; 70.9)	2934/3716	79.0 (77.7; 80.3)	<0.001
Skarbinski et al						
• Lindi, Tanzania	Health Facilities	507/637	79.6 (76.5; 82.7)	163/354	46.1 (40.9; 51.2)	<0.001
• Rufiji, Tanzania	Health Centre	1195/1463	81.7 (79.7; 83.7)	337/455	74.1 (70.0; 78.1)	<0.001
Stevenson et al						
• Western Kenya	School	595/1780	33.4 (31.2; 35.6)	2137/3742	57.1 (55.5; 58.7)	<0.001
**Individual ITN use**						
Mathanga et al						
• Malawi^d^	Health Centre	601/1637	36.7 (34.4; 39.1)	380/926	41.0 (37.9; 44.2)	0.0311
• Malawi^e^	Health Centre	943/1909	49.4 (47.2; 51.6)	1703/4565	37.3 (35.9; 38.7)	<0.001
Skarbinski et al						
• Lindi, Tanzania	Health Facilities	245/637	38.5 (34.7; 42.2)	78/354	22.0 (17.7; 26.4)	<0.001
• Rufiji, Tanzania	Health Centre	1042/1433	72. (70.4; 75.0)	241/455	53.0 (48.4; 57.6)	<0.001
**IRS coverage**						
Stevenson et al						
• Western Kenya	School	1216/1780	68.3 (66.2; 70.5)	2762/3742	73.8 (72.4; 75.2)	<0.001

#### Estimates of malaria morbidity

Six studies assessed the accuracy of estimates of malaria morbidity (Table 5) [41, 42, 44–46, 48]. All six studies evaluated estimates of *Plasmodium falciparum* prevalence rate (*Pf*PR) either by rapid diagnostic test (RDT), microscopy or polymerase chain reaction (PCR). In the studies where *Pf*PR was determined by microscopy, slides were double read [41, 44, 45] or single read by an expert microscopist [42]. As an additional measure, in two studies there was external quality control [41, 42], and in one study PCR was used to complement missing second reads and to disambiguate discordant species read results [45]. In three studies, estimates of *Pf*PR derived from EAGs were significantly higher than those of the population [45, 46, 48]. In one study [44], estimates of *Pf*PR derived from children attending health facilities for sick visits were not only concordant with population estimates but there was also concordance between results derived by microscopy and PCR. The accuracy of estimates *Pf*PR by RDT (Paracheck®, Orchid Biomedical Systems, India) derived from school children with circumferential distance was assessed in one study [48], and the estimate from this EAG remained consistently higher with increasing distance within the school catchment area.

**Table 5:** Comparison of estimates of coverage of malaria morbidity between EAGs and the population.

Control intervention coverage	Type of EAG survey	EAG survey		Population survey		Fisher’s exact p-value
		Events (n/N)	Percentage prevalence (95% CI)	Events (n/N)	Percentage prevalence (95% CI)	
*Pf*PR						
Gahutu et al						
• Butare, Rwanda (BS)	Health Centre	17/103	16.5 (9.3; 23.7)	61/545	11.2 (8.6; 13.8)	0.1286
• Butare, Rwanda (BS)	Hospital	10/101	9.9 (4.1; 15.7)	61/545	11.2 (8.6; 13.8)	0.8625
• Butare, Rwanda (PCR)	Health Centre	22/103	21.4 (13.4; 29.3)	88/545	16.2 (13.1; 19.2)	0.1994
• Butare, Rwanda (PCR)	Hospital^b,k^	15/101	14.9 (7.9; 21.8)	88/545	16.2 (13.1; 19.2)	0.8824
Hetzel et al						
• Momase and Highlands, Papua New Guinea (RDT)	Health Centre	402/1304	30.8 (28.3; 33.3)	199/1967	10.1 (8.8; 11.5)	<0.001
• Momase and Highlands, Papua New Guinea (RDT)	Health Centre	50/667	7.5 (5.5; 9.5)	50/1986	2.5 (1.8; 3.2)	0.001
Karyana et al						
• Mimika, Papua New Guinea (BS)	Health Centre	36848/253987	14.5 (14.4; 14.7)	290/3890	7.5 (6.6; 8.3)	<0.001
• Mimika, Papua New Guinea (BS)	Hospital	16895/168217	10.0 (9.9; 10.2)	290/3890	7.5 (6.6; 8.3)	<0.001
• Mimika, Papua New Guinea (BS)_	Hospital^d^	4195/17823	23.5 (22.9; 24.2)	290/3890	7.5 (6.6; 8.3)	<0.001
Mathanga et al						
• Malawi (2005, BS)	Health Centre	464/1516	30.6 (28.3; 32.9)	195/799	24.4 (21.3; 27.4)	0.0017
• Malawi (2008, BS)	Health Centre	247/1871	13.2 (11.7; 14.7)	607/4377	13.9 (12.8; 15.0)	0.4945
Oduro et al						
• Gambia (BS)	Health Centre	1088/4543	24.0 (22.7; 25.2)	487/3870	12.4 (11.3; 13.4)	<0.001
• Gambia (BS)	Health Centre	46/4101	1.1 (0.8; 1.4)	80/3716	2.2 (1.7; 2.6)	<0.001
Stevenson et al						
• Western Kenya	School	454/1780	25.5 (23.5; 27.5)	580/3742	15.5 (14.3; 16.7)	<0.001
APR						
Mathanga et al						
• Malawi (2005)	Health Centre	299/1636	18.3 (16.4; 20.2)	184/926	19.9 (17.3; 22.4)	0.3440
• Malawi (2008)	Health Centre	295/1909	15.5 (13.8; 17.1)	649/4461	14.6 (13.5; 15.6)	0.3557
Oduro et al						
• Gambia	Health Centre	440/4400	10.0 (9.1; 10.9)	283/3824	7.4 (6.6; 8.2)	<0.001
• Gambia	Health Centre^,^	317/3963	8.0 (7.2; 8.8)	127/3716	3.4 (2.8; 4.0)	<0.001
AbPR						
Oduro et al						
• Gambia	Health Centre	1122/3380	33.2 (31.6; 34.8)	736/3522	20.9 (19.6; 22.2)	<0.001
• Gambia	Health Centre	696/3362	20.7 (19.3; 22.1)	712/3391	21.0 (19.6; 22.4)	0.7875
Stevenson et al						
• Western Kenya	School	2536/4888	51.5 (49.2; 53.8)	1927/3742	51.5 (49.9; 53.1)	1.0000

BS = Blood slide

PCR = Polymerase chain reaction

RDT = Rapid diagnostic test.

Three studies assessed the ability of EAGs to measure changes in *Pf*PR as result of changes in coverage of interventions [42, 45] or seasonal transmission [41]. When data was collected before and one year after an ITN campaign in Papua New Guinea, the derived reduction in *Pf*PR by RDT in patients with a history of fever attending health facilities (absolute Risk Difference or RD = 23.3%, 95%CI 20.1%, 26.5%) was almost thrice that in the population (RD = 7.6%, 95%CI 6.1%, 9.1%) [45]. After a period of intense distribution of ITNs and a change in first line therapy of malaria from sulphadoxine-pyrimethamine to artemether-lumefantrine in Malawi, the reduction in *Pf*PR by malaria microscopy measured in children 6–30 months attending well child clinics (RD = 17.4%, 95%CI 14.6%, 20.2%) was higher than that in the same age strata in the population (RD = 10.5%, 95% CI 7.4%, 13.7%) [41], probably due to significantly higher EAG estimates in the first survey (Table 5). The study by Oduro et al [41] assessed the effect of seasonality on summary estimates *Pf*PR by malaria microscopy in all patients attending HFs in six ecologically diverse areas in Gambia, a country with intensely seasonal malaria transmission. In patients attending health facility regardless of cause, the reduction in *Pf*PR between the rainy season and the dry season (RD = 22.8%, 95%CI 21.6%, 24.1%) was almost twice that from the HF catchment population (RD = 10.4%, 95%CI 9.3%, 11.6%), probably due to the significantly higher estimates in the rainy/post-rainy season.

Two studies compared estimates of anaemia prevalence rate (APR) between EAGs and the population [41, 42]. In the study by Mathanga et al [42], estimates of APR from children attending well child clinics were not only concordant with values derived from the same age strata in the population but this metric in children attending well child clinics (RD = 2.8%, 95% CI 0.4%, 5.3%) accurately reflected the reduction in the population (RD = 5.3%, 95% CI 2.6%, 8.1%). The other study in Gambia assessed the impact of seasonality on estimates of APR derived from patients of all ages [41], and the difference between the rainy and dry season estimates from this EAG (RD = 2.0%, 95%CI 0.8%, 3.2%) was similar to that in the population (RD = 4.0%, 95%CI 3.0%, 5.0%) though both EAG estimates were consistently higher than population estimates (Table 5).

Two studies compared estimates of antibody prevalence between EAGs and the population [41, 48]. In the study in Gambia where malaria is intensely seasonal with one seasonal peak [41], the difference in Merozoite Surface Protein 1_19_ (MSP1_19_) seroprevalence between the seasons in the EAG (RD = 12.5%, 95%CI 10.4%, 14.6%) was higher than the population (RD = -0.1%, 95%CI -2.0%, 1.8%) due to overestimation of the population value in the rainy season (Table 5). In a moderately stable malaria transmission setting where there are two seasonal peaks of transmission (one major and the other minor), an assessment of AbPR using a number of antigens including Apical Membrane Antigen 1 (AMA1) and MSP1[48] in the month immediately after the major peak revealed that the estimate from school children (AbPR = 51.5%, 95% CI 49.2%, 53.8%) was concordant with that of the same age strata in the population (AbPR = 51.5%, 95% CI 49.9%, 53.1%, p = 1.000), and remained so with increasing distance within the school catchment area.

### Assessment of accuracy of EAG estimates

**Fig 2:**
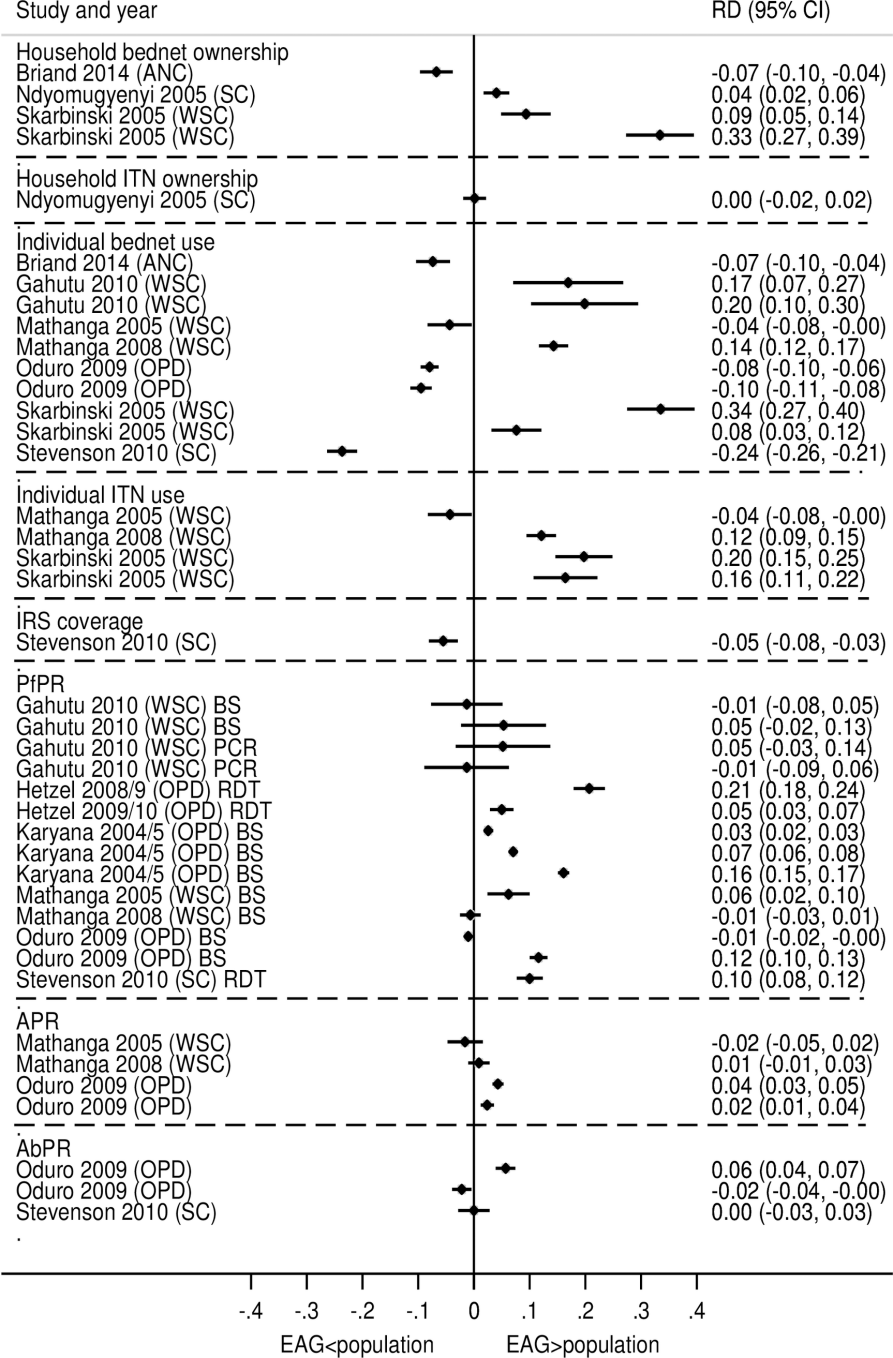
Absolute prevalence difference in estimates of standard malaria indicators. ANC = Antenatal Clinic OPD = All OPD SC = School children WSC = Well or sick child BS = Blood slide PCR = Polymerase chain reaction RDT = Rapid diagnostic test.

Except for the study by Ndyomugyenyi et al [47] were estimates of household ITN ownership derived from primary school children accurately represented population coverage (RD = 0, 95% CI -0.02, 0.02, p = 0.9759), estimates of control intervention coverage derived from EAGs were subject to widely varying degrees of inaccuracy (RD range: -0.24–0.42), with EAGs estimates more commonly but not exclusively over-estimating population values (Fig 2). In the two studies that assessed the accuracy of multiple indicators of intervention coverage [40, 42], estimates of related indicators usually had a similar level of inaccuracy (Fig 2). In the study by Mathanga et al [42], serial estimates of control intervention exhibited similar degree of bias in estimates of individual bed net and ITN use in the first survey but were markedly different in the subsequent survey (Fig 2). In the study by Skarbinski et al [40], the degree of accuracy in estimates of household bed net ownership, individual bed net and ITN use was the same for both well and sick child visits in Rufiji and ITN use in Lindi, whilst estimates of household bed net ownership and individual bed net use were much higher in Lindi (Table 2, Fig 2) indicting regional-specific bias (Fig 2).

Estimates of *Pf*PR were on average more consistent than estimates of intervention coverage (Fig 2). In the study by Gahutu et al [44], estimates of *Pf*PR by microscopy and PCR derived from EAGs at different health facility levels were concordant with population values (Fig 2). In the study by Mathanga et al [42], though serial estimates of *Pf*PR from children aged 6–30 months attending well child clinics accurately detected transmission reduction in the same age strata in the population, the estimate of P*f*PR from this EAG was slightly higher than that in the population in 2005 (RD = 0.06, 95% CI 0.02, 0.10, p = 0.002). Estimates of APR derived from EAGs in two studies [41, 42] were overall a more consistent estimation of population prevalence than *Pf*PR (Fig 2). The close approximation of EAG estimates of APR together with its accurate measurement of a reduction in population prevalence suggests that it is a good surrogate indicator for APR in the population [42]. Estimates of AbPR derived from EAGs were more accurate in the dry season in the Gambia [41], with rainy season estimates being higher than population estimates (RD = 0.12, 95% CI 0.10, 0.02, p<0.001).

Two of the studies attempted to measure the degree of inaccuracy or improve the precision of estimates by controlling for bias [40, 42]. After controlling for potential confounders (age in months, child’s sex, survey type and study area)in a multivariable analysis, in the study by Skarbinski et al [40], the adjusted odds ratio (aOR) between the health facility survey and the EAG survey for individual bed net use (aOR = 2.05, 95% CI 1.36, 3.08) and ITN use (aOR = 2.41, 95% CI 1.69, 3.44) still indicated an overestimation of population coverage. In the study by Mathanga et al [42], after adjusting for confounders in a multivariate analysis, parasitaemia in 2008 vs 2005 in children attending well child clinic (aOR = 0.31, 95% CI 0.22, 0.46) was equivalent to that in the same age strata in the population (aOR = 0.40, 95% CI 0.30, 0.52), and this was similar for anaemia (Hb<8.0d/dl) in this EAG (aOR = 0.85, 95% CI 0.65, 1.65) compared to the population (aOR = 0.74, 95% CI 0.59, 0.94).

**Fig 3:**
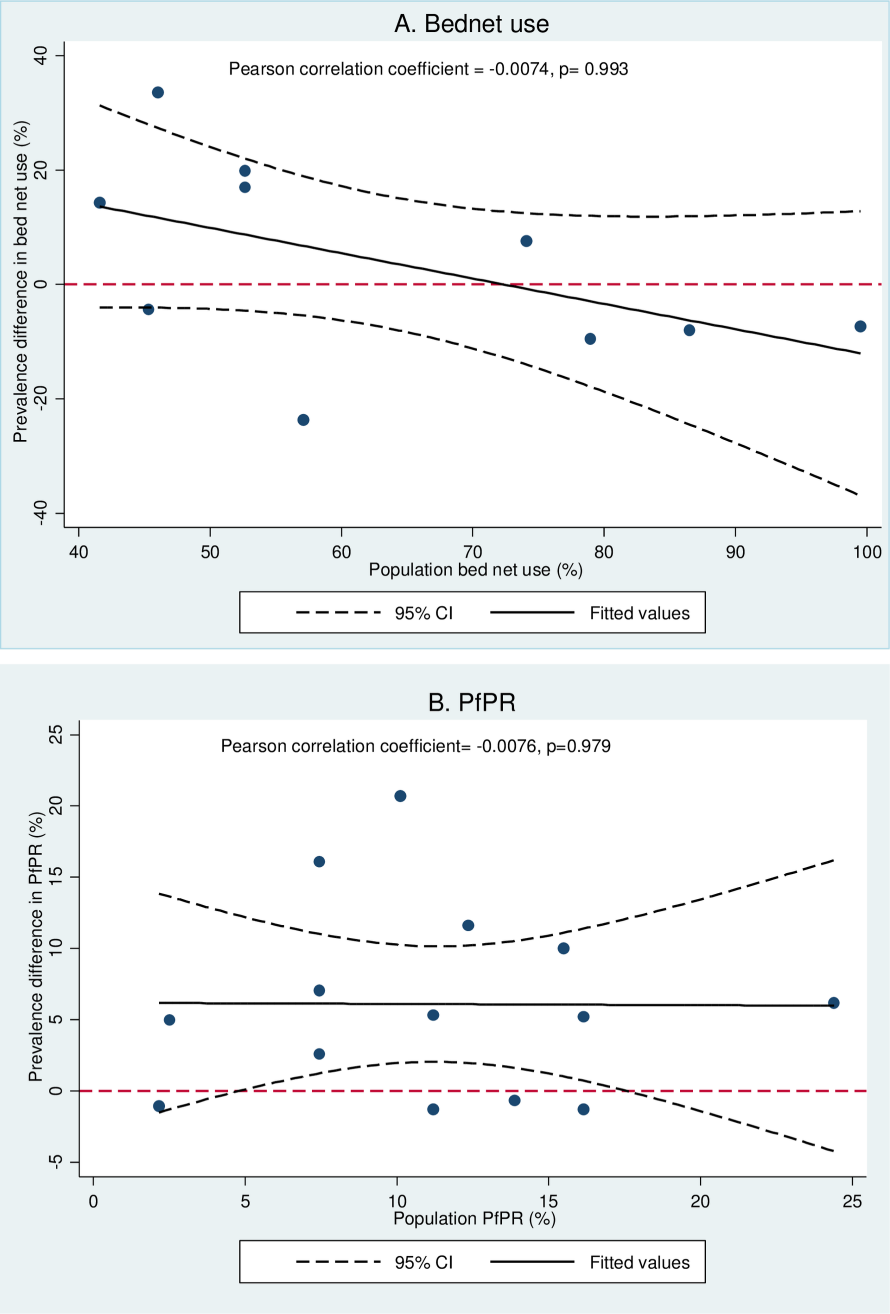
Prevalence difference of bed net use and *Pf*PR with population levels.

For EAG to guide control efforts, it should correctly classify the uptake of control interventions and malaria endemicity. The prevalence difference in bed net use suggested that EAG surveys overestimated population levels up to a certain point (population coverage of approximately 72%), after which they overestimated population values, but this trend was not statistically significant (p = 0.993) (Fig 3A). The prevalence difference in *Pf*PR overestimated population prevalence with increasing transmission (p = 0.979) (Fig 3B), but our assumptions are also limited by the fact that the studies included in this review only covered moderately stable and unstable endemic transmission intensities. Based on the classification of malaria endemicity from the *Pf*PR results, most of the EAG surveys (13/14) were concordant with that of the population (Table 6). During the post-ITN survey in Papua New Guinea [45], population *Pf*PR dropped to unstable endemic levels (*Pf*PR = 2.5%, 95%CI 1.8%; 3.2%) but was wrongly classified to be moderate stable by the EAG (PfPR = 7.5%, 95%CI 5.5%; 9.5%.).

**Table 6:** Relationship between the results of the classification of malaria endemicity between EAG and population sampling.

	Population		
EAG	Moderate stable	Unstable endemic	Total
Moderate stable	12	1	13
Unstable endemic	0	1	1
Total	12	2	14

## Discussion

Monitoring control progress is important to assess the effectiveness and coverage of malaria control programmes. Easy access group surveys are easier to conduct than population surveys and could provide accurate monitoring of control progress if the EAG sample is representative of our population stratum of interest [30, 33, 50–53]. Review of the available literature on EAGs suitable for district or sub-district surveillance of malaria control progress revealed a wide variation in the precision of estimates between and within studies, particularly for estimates of control intervention coverage. The small number of studies in this review shows how little effort has been made to explore the potential approach and settings for use of EAGs, probably due to the inherent assumption of bias in such opportunistic samples. Our study has potential limitations. Our search strategy may not have identified all the relevant papers or there may be other sources of grey literature that may have been missed. We phrased our search terms as simply as possible to allow a wider inclusion of possible papers and in this regard, we may have missed some papers with highly selective titles. The studies selected for the systematic review only included health facility (including ANC) and school surveys, and were from settings with moderate and intense stable malaria transmission, so our results may not be applicable to other EAGs or transmission settings. Our literature search was guided by categories of EAGs with historical evidence of use for malaria surveillance or which we theorized would be suitable for malaria surveillance at the district or sub-district level. This may have excluded publications on other potential EAGs. We limited our review to studies that compared EAG samples to populations samples of the same age or other at-risk stratum. Whilst this may improve the accuracy of EAG estimates of *Pf*PR, especially in moderate to severe transmission settings, this does not mean that EAGs could not be used to estimate control intervention coverage in any population stratum or *Pf*PR at the lower end of the transmission spectrum in other population at-risk strata. Given the pace of developments in analytical technics, this is an area where substantial gains can be made and we discuss this below.

### Dealing with bias in EAG surveillance

The main cause of bias in EAG surveillance is due to the selection of an unrepresentative sample of the population of interest. The opportunistic nature of the sampling frame in EAGs is inherently susceptible to selection bias when EAG sampling captures an unrepresentative subset of the population of interest. Particularly, if the reason for inclusion in the EAG sample is associated with the indicator of interest. For example, given the fact that those who are wealthier and more educated are more likely to attend health facilities, and have access to or use ITNs [54, 55], self-reported ITN possession and use from health facility surveys is likely to over-estimate ITN coverage in the population. This could be corrected using the verification rate measured from a small random sample of the catchment population. Also, the representativeness of estimates of *Pf*PR from health facilities is likely to be affected by the difference in transmission between malaria seasons, overall malaria transmission and the prevalence of non-malaria fevers. This could be limited by the use of EAGs excluding individuals coming for sick visits [56, 57] or prioritizing indictors that are less sensitive to short-term changes in transmission like AbPR [58, 59]. Population APR is also less sensitive to short term changes in transmission [60], but whether this makes it an appropriate indicator to measure changes in transmission is debatable. Though malaria is an important correlate of anaemia in children, the aetiology of anaemia is multifactorial and in particular the role of other infections, poor nutrition and the interaction between malaria and nutrition needs to be clarified [61]. Where there is a high probability of inclusion in the EAG sample, the difference in the estimates of an indicator measured from individuals who are and are not included in the EAG sample is likely to be less significant, and the EAG sample is more likely to be representative of the true situation in the population. For example, coverage rates of public health interventions were similar between vaccinated and unvaccinated children if population vaccine coverage was over 60% [62]. Most of the standard methods for analysis of data from convenience samples are based on the questionable assumption that selection bias can be exclusively ascribed to measured risk factors for malaria. Novel geostatistical methods have been recently developed to relax this assumption [63]. By combining data from unbiased gold-standard surveys and opportunistic samples, these methods are able to correct for the selection bias in the convenience samples that is induced by both measured and unmeasured risk factors. Though the aetiology of health facility access and utilization is multifactorial [64–69], health facility utilization follows a geographic pattern [70–73] and if this can be accurately measured through a small geospatially random sample of the population and accounted for in the model, will allow correction for bias and the production of accurate maps of control progress. Where point estimates are required, combining the EAG sample with a small and presumably far less expensive random sample of the population [74], the so-called hybrid sampling methodology will generate more accurate hybrid prevalence estimates. Pooling data from multiple EAGs in our area of interest is also likely to improve the precision of point estimates [47, 75].

Another cause of bias in EAG samples mainly affecting reported coverage of control interventions is social desirability bias. Survey respondents may answer questions in a manner they consider favourable to the interviewee leading to erroneously high self-reporting of coverage of control interventions [76]. This may be further compounded by the inability to directly validate the presence and use of household-level and individual control measures as in population surveys. Few studies have assessed the effect of social desirability bias on the effect of bed net use [77–80], and the wide range in verification rate of bed net use after self-report (60.9–96.2%) suggests variability in the effect of social desirability bias from setting to setting. Social desirability bias can be limited by modifying the standard MIS questionnaire [13] to include questions aimed at detecting and measuring social desirability bias so it can be directly accounted for in analysis [81], improving interviewee confidence by indicating the anonymity of their responses [81], or indirectly by correcting for the verification rate measured from a random sample of the catchment population.

### Use of EAG surveys to measure progress in malaria control

Before EAGs can be routinely used to measure malaria control progress, there are a few issues to address. Firstly, how much inaccuracy we are willing to tolerate? If the purpose of the survey is to measure trends in point estimates of control progress, some degree of inaccuracy is tolerable if EAG data displays similar trends to population data; as evidenced by the successful demonstration of transmission reduction from health facility surveys in some endemic countries [30, 33, 50–53], and increasing endorsement by WHO as a surveillance tool in different transmission settings and phases of control [1]. One study suggested that estimates of population *Pf*PR from health facility surveys might misclassify malaria endemicity at the lower end of the transmission spectrum [45], but the population in this study (i.e. all health facility attendees) may not be the most suitable to capture the most at-risk population at low transmission settings. When more accurate point estimates are required or accurate data is required over a large geographic area, pooling data from multiple similar EAGs [47, 75] or hybrid sampling methodology [74] may improve precision. If the purpose of the survey is to measure changes in the the geospatial distribution of uptake of control interventions and transmission intensity, to identify areas of low intervention coverage and potential hotspots respectively for targeted control intervention delivery; the smaller sized EAG catchment areas compared to community-based surveys [70–73] means the maps derived using EAG sampling will not be consistent with those derived using community-level data and would require geospatial statistical methods to correct for bias [63].

Secondly, are EAGs surveys more cost effective than standard approaches? Because of the ease of EAG sampling, conducting an EAG survey should theoretically be cheaper than a population survey in the same catchment area. Reports from school surveys in Kenya seem to suggest that the financial cost of school surveys is less than half that of a household survey [9, 11]. Though a detailed economic costs analysis of school surveys has not been done in comparison to those from household surveys, and the lower financial costs has not been validated in other EAGs; the decreased expenditure on personnel, transportation and communication in school surveys compared to household surveys suggest that surveillance in EAGs is likely to be more cost-effective [9].

Thirdly, when are EAG surveys most likely to be beneficial i.e. to complement malaria programmatic efforts? At moderate to high malaria transmission intensity, surveillance systems rely on passive surveillance (e.g. HMISs) supplemented by large serial populations surveys (e.g. MISs), with data reported at the national, regional and sometimes district level. Surveillance in EAGs in such settings will be beneficial in providing more detailed sub-district estimates from “problematic” districts with poor control progress compared to national average, estimates from hard-to-reach communities (e.g. opportunistic surveys during MDA) who would otherwise not be covered by population surveys, or when data is required to assess at-risk stratum specific control interventions (e.g. ANC and delivery surveys to assess the impact of Intermittent Preventive Treatment in pregnancy or IPTp). These EAG surveys should be carried out at the same time as population surveys i.e. every 2–3 years, so that the estimates can be interpreted within the context of a wider perspective of population control progress. As transmission intensity falls and we approach the elimination phase, reorientation of programmatic efforts are required to identify hotspots [8] and special high-risk populations [82, 83] both of which serve as reservoirs of infection that should be targeted for malaria elimination. Population surveys become less logistically attractive and less practical given the fact that more regular (e.g. quarterly) local (sub-district) level data is required on control progress. Surveillance in EAGs becomes more attractive as a more sustainable method of surveillance including the high-risk groups (e.g. rural community market surveys at border crossings).

Finally, how do we integrate surveillance in EAGs with current control strategies? EAG surveillance can provide timely data of reasonable accuracy on control progress that reflect local variation at the district or sub-district level, and is complementary to national community-based surveys like MISs [13]. EAG surveys can provide a means of rapid assessment of areas known to have poor coverage or key population risk-strata. The ease of sampling and low costs allows more frequent or even continuous surveys providing timely data and encouraging reactive targeted control. EAG surveillance in health facilities may have a motivational impact on health workers at the district and sub-district level through the provision of continuous locally appropriate data on intervention coverage and malaria transmission, and its flexibility allows it to adapt to new programmatic needs over time. Sufficient person-time is however needed for successful data acquisition in health facilities and to ensure no duplication with recurrent data collection. Implementing and scaling up EAG surveillance will require minimal reorientation and structuring of the health system, including determining which health facility personnel should be dedicated to malaria surveillance, and some preparation and buy-in is required by both national and global health players.

## Conclusions

This review describes the previous experiences with the validation of estimates of malaria control progress from different EAGs and highlights the potential of surveillance in EAGs as a complementary approach to current surveillance systems. The utility of an EAG for routine surveillance of progress in malaria control at the district or sub-district programmatic level will be driven by several factors including whether serial point estimates or more precise geospatial distribution is required, the degree of precision accepted, the desired population of interest (e.g. at-risk groups), and the resources available for surveillance (both financial and otherwise).The low cost of EAG surveillance, its flexibility and potential to offer locally applicable timely estimates of control which could improve programmatic responses suggest that further validation and optimization is required.

## Supporting information

S1 Document PRISMA 2009 checklist. (DOC)
